# Long term condition morbidity in English general practice: a cross-sectional study using three composite morbidity measures

**DOI:** 10.1186/s12875-016-0563-3

**Published:** 2016-11-29

**Authors:** Charlotte Weston, Alexander Gilkes, Stevo Durbaba, Peter Schofield, Patrick White, Mark Ashworth

**Affiliations:** Division of Health and Social Care Research, Department of Primary Care and Public Health Sciences, King’s College London, Addison House, Guy’s Campus, London, SE1 1UL UK

**Keywords:** Primary care, Long term conditions, Multimorbidity, Patient experience, Secondary care utilisation

## Abstract

**Background:**

The burden of morbidity represented by patients with long term conditions (LTCs) varies substantially between general practices. This study aimed to determine the characteristics of general practices with high morbidity burden.

**Method:**

Retrospective cross-sectional study; general practices in England, 2014/15. Three composite morbidity measures (MMs) were constructed to quantify LTC morbidity at practice level: a count of LTCs derived from the 20 LTCs included in the UK Quality and Outcomes Framework (QOF) disease registers, expressed as ‘number of QOF LTCs per 100 registered patients’; the % of patients with one or more QOF LTCs; the % of patients with one or more of 15 broadly defined LTCs included in the GP Patient Survey (GPPS). Determinants of MM scores were analysed using multi-level regression models. Analysis was based on a national dataset of English general practices (*n* = 7779 practices); GPPS responses (*n* = 903,357); general practice characteristics (e.g. list size, list size per full time GP); patient demographic characteristics (age, deprivation status); secondary care utilisation (out-patient, emergency department, emergency admission rates).

**Results:**

Mean MM scores (95% CIs) were: 57.7 (±22.3) QOF LTCs per 100 registered patients; 22.8% (±8.2) patients with a QOF LTC; 63.5% (±11.7) patients with a GPPS LTC. The proportion of elderly patients and social deprivation scores were the strongest predictors of each MM score; scores were largely independent of practice characteristics. MM scores were positive predictors of secondary care utilization and negative predictors’ access, continuity of care and overall satisfaction.

**Conclusions:**

Wide variation in LTC morbidity burden was observed across English general practice. Variation was determined by demographic factors rather than practice characteristics. Higher rates of secondary care utilisation in practices with higher morbidity burden have implications for resource allocation and commissioning budgets; lower reported satisfaction in these practices suggests that practices may struggle with increased workload. There is a need for a readily available metric to define the burden of morbidity and multimorbidity in general practice.

## Background

In the last two decades there has been an increasing focus in primary care on the management of long term conditions (LTCs) with a parallel reduction in presentation of acute self-limiting conditions to general practitioners (GPs) [1]. Approximately one in six patients registered with a GP have one or more LTCs that feature in the Quality and Outcomes Framework (QOF), the pay-for-performance scheme which rewards GPs for achieving clinical targets in patients with LTCs; approximately one third of all GP consultations are related to LTCs [2].

Huntley et al. performed a systematic review to identify and compare measures of morbidity burden suitable for use in primary care research. These included proposed measures of morbidity such as simple disease counts or weightings according to the perceived severity, mortality or resource utilization of specific LTCs [3]. The choice of measure used was found to be based on the suitability of the measure for the data available as well as the outcome of interest. None of the existing measures are readily applicable to a national sample of GP practices in England.

Few studies have measured the variation in the burden of LTC morbidity at practice level in primary care, primarily due to the lack of consensus about an optimal composite indicator for measuring morbidity. Fortin et al. conducted a systematic review of 21 international studies of LTC or multimorbidity prevalence in primary care and found wide variation in prevalence estimates mainly among those aged over 75 years in whom estimates of variation differed by up to 95%, although much of this difference was attributed to methodological inconsistencies such as variation in the number of included LTCs ranging from five to 185 [4]. Differing methods of collecting data also contributed to variance in prevalence estimates and using combined inputs of data from self-reported patient data and physician reports resulted in more reliable estimates. Socioeconomic deprivation is related to the prevalence of LTCs with higher prevalence of cardiovascular disease, chronic obstructive pulmonary disease and diabetes in deprived populations [5].

In the UK the prevalence of LTC morbidity in primary care is known to be related both to GP workload and some aspects of secondary care costs. Glynn et al. demonstrated an almost linear relationship between the number of LTCs per patient and out-patient appointment attendance [6].

LTC morbidity and multimorbidity are associated with reduced quality of life scores; however, few studies have directly addressed patient satisfaction in the presence of LTCs [7]. Fan et al. found self-reported ability of coping with an LTC had the biggest impact on satisfaction with their primary health care provider [8]. Paddison et al. looked at a subset of patients who reported poor health based on data from the GP Patient Survey (GPPS) and found GP communication, along with continuity of care, were the strongest drivers of overall satisfaction [9].

Our principal aim was to determine the characteristics of general practices which have a high burden of LTC morbidity. We had three secondary aims. Firstly, to devise and test morbidity measures applicable to English primary care and designed to capture the burden of LTC morbidity. Secondly, to determine the variation in LTC morbidity levels between GP practices. Thirdly, to assess the impact of LTC morbidity on secondary care utilization and overall patient satisfaction. By exploring several aspects of LTC morbidity, we hoped to develop a composite picture of the burden of LTC morbidity in primary care.

## Method

### Study design

We conducted a retrospective cross sectional study using practice-level data in England. Data were obtained from publicly available national datasets.

The QOF is the voluntary annual incentive and reward programme based on the achievement of pre-specified targets and the construction of disease registers for specified LTCs. These disease registers lend themselves to studies of morbidity and currently include 20 LTCs (see below). We obtained QOF data for all practices in England, 2014/15 (*n* = 7779) covering almost all registered patients in England [10].

Additional practice data were obtained, including workforce information, practice characteristics such as registered patient list size, list size per full-time equivalent GP, and patient age distribution [11]. Estimates of the proportions of patients from ethnic minority groups (African-Caribbean and South Asian populations) and the level of social deprivation (patient level adjusted IMD-2010 scores) were obtained for the location of each practice, based on Lower Layer Super Output Area (LLSOA) data for the practice postcode [12].

We obtained 2014/15 secondary care utilisation data for general practices [11]. These data included rates for Accident and Emergency (A&E) attendance, emergency hospital admissions, and out-patient attendances.

Patient satisfaction data were obtained from the GPPS, a nationwide patient experience survey administered by Ipsos MORI [13]. In total, 2.6 million questionnaires were distributed in 2014/15 and 903,357 (34%) responses were received. Responses were scored either as dichotomous variables (yes or no) or 4 to 5 point Likert scales.

### Exclusion criteria

Practices were excluded from the dataset if they had a list size per full time equivalent (FTE) GP under 500 or over 5000, fewer than 750 patients or missing list size data. These exclusion criteria were based on the assumption that these were likely to be atypical practices. Also to limit any measurement bias in patient experience we excluded any practices that had less than 100 GPPS responses. Our analysis was conducted on the remaining 7556 (97%) practices.

### Composite measures of long term condition morbidity

We constructed three proxy measures in order to determine the burden of LTC morbidity in each general practice:

Morbidity measure 1: a count in each practice of LTCs based on the 20 LTCs included in the UK Quality and Outcomes Framework (QOF) disease registers, expressed as ‘number of QOF LTCs per 100 registered patients’ [10]. Measure 1 represents a practice level count of selected LTCs (Table 1).

**Table 1:** Long Term Conditions (LTCs) included in each morbidity measure

Morbidity measure 1 [number of QOF LTCs per 100 registered patients] (Mean prevalence)^a^	Morbidity measure 2 [% of patients with one or more QOF LTCs]	Morbidity measure 3 [% of patients with one or more GPPS LTCs]
Coronary Heart Disease (3.29%) Peripheral arterial disease (0.63%) Stroke or TIA (1.7%) Hypertension (14.05%) Diabetes (5.31%) Chronic Obstructive Pulmonary Disease (1.89%) Chronic Kidney Disease (3.26%) Asthma (5.97%) Schizophrenia & bipolar affective disorder (0.91%) Atrial fibrillation (1.59%) Dementia (5.31%) Depression (5.72%) Epilepsy (0.63%) Heart failure (0.73%) Learning difficulty (0.46%) Osteoporosis (0.06%) Palliative care (0.32%) Cancer (2.22%) Rheumatoid arthritis (0.61%) Obesity (7.61%)	Coronary Heart Disease Peripheral arterial disease Stroke or TIA Hypertension Diabetes Chronic Obstructive Pulmonary Disease Chronic Kidney Disease Asthma Schizophrenia & bipolar affective disorder	Dementia or Alzheimer’s Angina or long term heart condition Arthritis or long term joint condition Asthma or long term chest condition Blindness or severe visual impairment Cancer in last 5 years Deafness or severe hearing impairment Diabetes Epilepsy Hypertension Kidney or liver disease Learning difficulty Long term back problem Long term mental health problem Long term neurological problem

^a^prevalence as a percentage of all registered patients, derived from QOF disease registers

Morbidity measure 2: the percentage of patients in each practice with one or more QOF LTCs. Although QOF data are aggregated and not available at patient-level, one of the QOF indicators (‘Smoking2’) [10] provides data on the number of patients with one or more of a sub-set of 9 LTCs (Table 1). The denominator of this indicator was used to calculate the percentage of patients with the selected LTCs. Measure 2 represents the proportion of patients with one or more selected QOF LTCs.

Morbidity measure 3: the percentage of GPPS respondents in each practice who report that they have one or more LTCs. The GPPS asks patients if they have any one of 15 broadly defined LTCs (Table 1) [13]. Measure 3 represents the proportion of patients with one or more selected GPPS LTCs.

### Statistical analysis

We used univariable methods to describe practice-level morbidity based on the three composite morbidity measures and also to describe practice and demographic variables.

Multivariable analysis was used to explore predictors of morbidity and the role of morbidity in determining secondary care utilisation and patient satisfaction.

Firstly, we constructed multiple linear regression models to determine practice level factors associated with high burdens of morbidity. The three composite morbidity measures were used as the outcome variables in separate regression models. We included the following practice-level predictor variables: proportion of patients aged 45–64 years, proportion of patients aged 65–74 years, proportion of patients aged 75–84 years and proportion of patients aged over 85 years, patient level index of multiple deprivation (IMD-2010) score, proportion of people of African/Caribbean and South Asian ethnicity, list size per FTE GP, QOF clinical domain points scored and GP training practice status.

Secondly, we constructed multiple linear regression models to determine the role of morbidity burden as a predictor of secondary care utilisation. We constructed separate models for each of the three secondary care measures (as outcome variables) and for each composite morbidity measure (included individually in the model as a predictor variable).

Finally, we constructed regression models to determine the role of morbidity as a predictor of patient satisfaction. We focused on four separate GPPS domains, namely access to care, communication, continuity of care and overall satisfaction using a previously described methodology, which produced seven scores from these four domains. These domains were chosen as they have previously been validated for use in exploring patient experience in LTCs [14]. We constructed a value for a ‘positive response’ in the GPPS by summating the proportion of patients reporting either of the two most positive responses (for instance, ‘very good’ or ‘good’). We constructed separate models for each of the seven satisfaction scores (as outcome variables) and for each composite morbidity measure (included individually in the model as a predictor variable).

To test for multi-collinearity the variance inflation factor (VIF) was calculated and any variables exceeding VIF > 10 were excluded. A multi-level regression approach allowed us to adjust for clustering at Clinical Commissioning group (CCG) level and at practice level using a random intercept model to account for specific effects at these levels. Regression model assumptions were checked graphically for each regression equation.

Standardised beta coefficients, with 95% confidence intervals, were used to examine the relative strength of the various predictors of morbidity burden within practices and also the relative effect of morbidity burden on health service utilization and GPPS scores. STATA software version 14 (Statacorp, Texas) was used for all statistical analysis.

## Results

### Sample characteristics

The demographic details and characteristics of practices included in our sample are summarised in Table 2.

**Table 2:** National sample of general practices in England, 2014/2015. (*n* = 7556)

Practice or population characteristic	Mean (SD), or n
GP training practice	*n* = 1911
QOF clinical domain score	411 (31.5)
Practice list size	7355 (4370)
Practice list size per full-time equivalent GP	1805 (686)
Registered patients aged 45–64 years (%)	25.4 (4.3)
Registered patients aged 65–74 years (%)	9.2 (3.4)
Registered patients aged 75–84 years (%)	5.4 (2.1)
Registered patients aged ≥85 years (%)	2.2 (1.1)
Index of Multiple Deprivation score (IMD-2010)	26.2 (17.4)
Population Ethnicity: South Asian (%)	8 (13)
Population Ethnicity: African or Caribbean (%)	4 (6)

### Long Term Condition morbidity prevalence values in general practices

Practices in the highest fifth centile for the QOF-based composite morbidity measures had more than double the morbidity burden of those in the lowest fifth centile (Table 3). Morbidity measure 3, a broader definition of morbidity, showed less variation.

**Table 3:** Mean values of each morbidity measure

	Morbidity measure 1: number of QOF LTCs per 100 registered patients	Morbidity measure 2: % of patients with one or more QOF LTCs	Morbidity measure 3: % of patients with one or more GPPS LTCs
Mean value (5th–95th centiles)	56.7 (35.1–76.7)	22.6% (14.3–29.5)	63.5% (51.8–73.8)

The strongest predictors of all three composite measures of morbidity were the proportion of patients aged 65–74 years and 75–84 years and the IMD score (Table 4). Training practice status, list size per FTE GP and African Caribbean or South Asian ethnicity were weak negative predictors of morbidity burden.

**Table 4:** Practice and demographic predictor variables for the three composite morbidity measures

Predictor variables: Beta coefficients for variables that were significantly associated with higher morbidity burden (*P* < 0.05); n/s denotes not significant			
	Morbidity measure 1:	Morbidity measure 2:	Morbidity measure 3:
Registered patients aged 45–64 years (%)	0.17	0.23	0.15
Registered patients aged 65–74 years (%)	0.34	0.43	0.15
Registered patients aged 75–84 years (%)	0.27	0.38	0.33
Registered patients aged ≥85 years (%)	0.22	0.02	n/s
IMD score	0.51	0.35	0.62
QOF points (clinical domain indicators)	0.11	0.04	n/s
Training Practice status	n/s	−0.16	n/s
Proportion of population of African/Caribbean ethnicity	−0.13	−0.03	−0.15
Proportion of population of South Asian ethnicity	−0.04	0.04	−0.10
List size per FTE GP	n/s	n/s	−0.03

### LTC morbidity burden as a predictor of health service utilization

Each composite morbidity measure was a significant predictor of health service utilization (Table 5). Clustering at PCT level explained less than 5% of the variation in secondary care utilization (detailed results are available from the authors).

**Table 5:** LTC morbidity burden as a predictor of health service utilisation

Beta coefficient (all p <0.05)			
	Morbidity measure 1	Morbidity measure 2	Morbidity measure 3
Emergency admissions per 1000 (crude rate)	0.28	0.35	0.21
A + E attendance per 1000 (crude rate)	0.30	0.38	0.21
Outpatients attendance per 1000 (crude rate)	0.27	0.47	0.14

### LTC morbidity burden as a predictor of patient satisfaction

Higher morbidity levels, as represented by higher composite Morbidity measure scores, were associated with lower satisfaction with access, continuity of care and lower overall satisfaction. However, higher morbidity was associated with greater reported satisfaction with nursing care (Table 6). There was no consistent relationship between the composite morbidity measures and reported satisfaction with doctor care or reception communication. Clustering at PCT level explained less than 5% of the variation in patient satisfaction (detailed results are available from the authors).

**Table 6:** B coefficients between GPPS domain score and Morbidity Score

GPPS question	Morbidity measure 1	Morbidity measure 2	Morbidity measure 3
B coefficients (*P* < 0.05) ; N/S denotes non-significant			
Access (q3)	−0.08	−0.29	−0.21
Access (q18)	−0.05	−0.19	−0.12
Continuity of care (q9)	−0.17	−0.37	−0.10
Communication (Reception) (q4)	n/s	n/s	n/s
Communication (Doctor) (q21a-e)	n/s	n/s	n/s
Communication (Nurse) (q23a-e)	0.14	0.40	0.13
Overall experience (q28)	n/s	n/s	−0.04

## Discussion

### Summary

We have constructed three composite LTC morbidity measures, two based on QOF disease registers and one based on GP Patient Survey responses, which can readily be applied to general practices in England. These measures have demonstrated substantial inter-practice variation in the burden of LTC morbidity.

Higher LTC morbidity burden was associated with higher levels of all aspects of secondary care utilisation included in our study. For most of the GPPS patient satisfaction measures, especially the global measure of ‘overall satisfaction’, higher LTC morbidity burden was associated with lower reported patient satisfaction. Overall satisfaction with nursing care, however, was greater in practices with a higher LTC morbidity burden. This may be a reflection of the greater role of practice nurses in the management of patients with LTCs and subsequent positive experiences of patients whose LTCs are managed by the nurse [15].

### Comparison with existing literature

Our findings that morbidity prevalence was positively related to the proportion of patients in increasing age ranges and social deprivation are congruent with other studies examining the link between age, deprivation and morbidity prevalence [5, 16]. The 45–64 year old age category showed a positive association with morbidity burden, consistent with the findings of Barnett et al. that the absolute number of patients with multimorbidity was greatest in those aged under 65 years old [16]. Morbidity prevalence increased consistently with age until 85 years, when the association plateaued, supporting the findings of other studies demonstrating an S-shaped curve for the association between age and LTC prevalence*”* [4]. Higher levels of morbidity are associated with increased workload in primary care and referral rates to secondary care and there is an increased likelihood of needing to use health services with increasing numbers of long term conditions [17].

In our study, practices with higher morbidity burdens demonstrated higher levels of satisfaction in the nursing care domain but no significant relationship was found with doctor care satisfaction scores. LTC care provision has shifted from doctors to nurses and this shift appears to have resulted in greater patient satisfaction. Evidence of enhanced patient satisfaction with nurse consultations has also been reported for ‘same day’ consultations, usually involving acute self-limiting illnesses; specific factors which improved satisfaction were longer consultation times and being given more information [18].

The GPPS asks if patients have a preferred GP and responses to this question are interpreted as a marker of continuity of care [19]. We found that practices with higher burdens of morbidity have lower rates of reported ‘preferred GP’, indicating that patients with LTCs in these practice may experience difficulty in obtaining consultations with a specific GP. Alternatively, patients in practices with higher morbidity may be trading off a preference for continuity of care in favour of more rapid access to primary care. Other studies have demonstrated the importance of continuity of care to patients with LTCs and emphasised the association between continuity of care and improved care outcomes including lower hospital admission rates and better preventative services [20, 21]. Studies have demonstrated an inconsistent relationship between access and patient satisfaction; a recent systematic review found that high levels of access were not consistently associated with changes in clinical outcomes, patient satisfaction, or health service utilization among patients with long term conditions [22]. However, other studies have shown improved continuity of care and patient satisfaction amongst patients who registered at practices with higher levels of access to appointments [23, 24].

### Strengths and limitations

The nature of a practice-level study meant that our findings could have been subject to bias arising from the ecological fallacy. Without access to patient-level data, we cannot demonstrate causal relationships. However, our three separate morbidity measures were consistent and plausible in demonstrating the relationship between practice level morbidity burden, health service utilization and patient satisfaction. The analysis of morbidity burden would be enhanced by access to consultation rate data to illustrate the potential impact of morbidity on workload in primary care.

Each measure of morbidity was limited by the number of LTCs that were included, which may have potentially limited the application of each morbidity measure for international comparisons. Morbidity Measures 2 and 3 were measures of the proportion of patients with one or more LTCs, one based on a narrow set of QOF defined LTCs classified by GPs and one based on more broadly defined LTCs selected by patients. Fortin et al. found that studies that considered 12 or more LTC diagnoses had little variation in morbidity prevalence and should suffice to measure multimorbidity accurately [4]. Harrison et al. further elaborated that this was only likely to be sufficient if using a definition of multimorbidity as the “co-occurrence of two or more chronic conditions within one person without defining an index chronic condition” [25].

The low response rate (36%) to the national GPPS may diminish the generalisability of findings to the primary care population and practices. To counter this, since 2011, Ipsos MORI have applied weightings to questionnaire distribution in order to reduce non-response bias. Furthermore, non-response bias has not been found to be associated with patients with LTCs [26].

## Conclusions

The development of LTC morbidity measures using routinely available primary care data is important in terms of understanding variation between practices, their utilisation of secondary care services and differences in reported patient satisfaction. Identifying practices with high morbidity prevalence is likely to have implications for resource allocation, commissioning budgets and the setting of notional practice-based budgets for secondary care [17]. We have demonstrated that composite indicators of the burden of LTC morbidity in each general practice can readily be constructed using QOF or GPPS data. ‘Morbidity measure 2’ may be suitable for development into a primary care indicator representing the proportion of patients with one of more LTCs, although it is limited to nine separate but high prevalence LTCs (See Table 1).

Our study has health economic implications which we have not explored. Further research is needed on the increased resource requirements of LTCs in primary care both within general practices (increased consultation rate; increased consultation duration) and in secondary care (from commissioning budgets). Furthermore, if care for patients with multimorbidity continues to shift from secondary into primary care, further research is needed into the investment required in order to manage the additional burden of LTC morbidity more efficiently [27].
